# Exploring the neural basis of non-invasive prehabilitation in brain tumour patients: An fMRI-based case report of language network plasticity

**DOI:** 10.3389/fonc.2024.1390542

**Published:** 2024-05-17

**Authors:** Leonardo Boccuni, Alba Roca-Ventura, Edgar Buloz-Osorio, David Leno-Colorado, Jesús Martín-Fernández, María Cabello-Toscano, Ruben Perellón-Alfonso, Jose Carlos Pariente Zorrilla, Carlos Laredo, Cesar Garrido, Emma Muñoz-Moreno, Nuria Bargalló, Gloria Villalba, Francisco Martínez-Ricarte, Carlo Trompetto, Lucio Marinelli, Matthew D. Sacchet, David Bartrés-Faz, Kilian Abellaneda-Pérez, Alvaro Pascual-Leone, Josep María Tormos Muñoz

**Affiliations:** ^1^ Institut Guttmann, Institut Universitari de Neurorehabilitació adscrit a la UAB, Barcelona, Spain; ^2^ Universitat Autònoma de Barcelona, Cerdanyola del Vallès, Spain; ^3^ Department of Conegliano, Scientific Institute IRCCS E. Medea, Treviso, Italy; ^4^ Fundació Institut d’Investigació en Ciències de la Salut Germans Trias i Pujol, Barcelona, Spain; ^5^ Departament de Medicina, Facultat de Medicina i Ciències de la Salut, Institut de Neurociències, Universitat de Barcelona, Barcelona, Spain; ^6^ Department of Neurosurgery, Hôpital Gui de Chauliac, Montpellier, France; ^7^ Department of Neurosurgery, Hospital Universitario Nuestra Señora de Candelaria, Santa Cruz de Tenerife, Spain; ^8^ Universidad de La Laguna, San Cristóbal de La Laguna, Spain; ^9^ Institut d’Investigacions Biomèdiques August Pi i Sunyer (IDIBAPS), Barcelona, Spain; ^10^ Magnetic Resonance Image Core Facility (IDIBAPS), Barcelona, Spain; ^11^ Neuroradiology Section, Radiology Department, Diagnostic Image Centre, Hospital Clinic of Barcelona, University of Barcelona, Barcelona, Spain; ^12^ Centro de Investigación Biomédica en Red de Salud Mental (CIBERSAM), Instituto de Salud Carlos III, Barcelona, Spain; ^13^ Department of Neurosurgery, Hospital del Mar, Barcelona, Spain; ^14^ Department of Neurosurgery, Vall d’Hebron Hospital, Universitat Autònoma de Barcelona, Barcelona, Spain; ^15^ Department of Neuroscience, Rehabilitation, Ophthalmology, Genetics, Maternal and Child Health, University of Genova, Genova, Italy; ^16^ IRCCS Ospedale Policlinico San Martino, Genova, Italy; ^17^ Meditation Research Program, Department of Psychiatry, Massachusetts General Hospital, Harvard Medical School, Boston, MA, United States; ^18^ Athinoula A. Martinos Centre for Biomedical Imaging, Department of Radiology, Massachusetts General Hospital, Harvard Medical School, Boston, MA, United States; ^19^ Hinda and Arthur Marcus Institute for Aging Research and Deanna and Sidney Wolk Centre for Memory Health, Hebrew Senior Life, Boston, MA, United States; ^20^ Department of Neurology, Harvard Medical School, Boston, MA, United States; ^21^ Centro de Investigación Traslacional San Alberto Magno, Universidad Católica de Valencia San Vicente Mártir, Valencia, Spain

**Keywords:** brain tumour, prehabilitation, neurorehabilitation, neuromodulation, fMRI, case report

## Abstract

Primary brain neoplasms are associated with elevated mortality and morbidity rates. Brain tumour surgery aims to achieve maximal tumour resection while minimizing damage to healthy brain tissue. Research on Neuromodulation Induced Cortical Prehabilitation (NICP) has highlighted the potential, before neurosurgery, of establishing new brain connections and transfer functional activity from one area of the brain to another. Nonetheless, the neural mechanisms underlying these processes, particularly in the context of space-occupying lesions, remain unclear. A patient with a left frontotemporoinsular tumour underwent a prehabilitation protocol providing 20 sessions of inhibitory non-invasive neuromodulation (rTMS and multichannel tDCS) over a language network coupled with intensive task training. Prehabilitation resulted in an increment of the distance between the tumour and the language network. Furthermore, enhanced functional connectivity within the language circuit was observed. The present innovative case-study exposed that inhibition of the functional network area surrounding the space-occupying lesion promotes a plastic change in the network’s spatial organization, presumably through the establishment of novel functional pathways away from the lesion’s site. While these outcomes are promising, prudence dictates the need for larger studies to confirm and generalize these findings.

## Introduction

Brain tumours are characterized by high mortality rate, severe disability, and burden for the healthcare system. A systematic analysis from the Global Burden of Disease Study outlined that, in 2016 alone, the global incidence of primary brain and central nervous system tumours was 330,000 new cases and 227,000 deaths (1). The overall 5-year survival rate for malignant brain tumours is 36% (2), despite advancements in the field of neurosurgery, radiotherapy, and chemotherapy. Patient’s survival is associated with both the extent of tumour resection and postoperative neurological deficits, so that the best outcomes are expected for patients with gross total resection and no worsening of symptoms (3, 4). However, massive resection and preserved functionality are often conflicting goals, posing neurosurgeons in the dilemma of finding a cost-benefit compromise.

One promising approach is Neuromodulation-Induced Cortical Prehabilitation (NICP) (5). NICP aims at leveraging neuroplastic changes before surgery, by performing conditioning sessions over several consecutive days or weeks. This neuroplasticity-based paradigm holds the potential to modulate brain connectivity and activity, facilitating the transfer of functional activity from one brain region to another. The goal of this process is to broaden safe functional margins for excision, to maximize tumour eradication while at the same time preserving neurological status. So far, publications on NICP account for four case reports and one case series, totalling only eight patients (6–10). A common element of all NICP studies is a two-step process, the first step being the ‘virtual lesion’ of areas considered at risk of being compromised during neurosurgery; and the second step being the promotion of brain activity of alternative brain resources, while the targeted area has been inhibited.

The accomplishment of the first step (i.e., virtual lesion), can be performed invasively, by means of extra-operative continuous high-frequency cortical electrical stimulation (7–9), or non-invasively, for instance, by transcranial magnetic stimulation (TMS) (6, 10). Invasive neuromodulation has been investigated in two case reports (7, 9) and a case series (8), showing consistent patterns of neural reorganization studied through functional magnetic resonance imaging (fMRI). However, invasive techniques required two surgeries and came at the cost of high rate of complications such as infections and seizures (8). Non-invasive neuromodulation was investigated in two case reports by Barcia et al. (6) and Dadario et al. (10). However, task-evoked brain reorganizations were not significant (6) or not reported (10).

The second step (i.e., enhancement of activity for alternative brain areas) is achieved by training the function at risk of being compromised. Such intervention is performed during and/or immediately after inhibition of targeted peritumoural areas, in a condition where the brain is supposedly constrained to recruit alternative pathways within the same functional network. Type and amount of training varied greatly among protocols, from no training (10) up to six hours a day (9).

Given the limited number of studies, the complex nature of the interventions, and the diversity of protocols, the impact of non-invasive NICP interventions at the neuroimaging level, as well as the underlying neurobiological mechanisms responsible for these changes, remains largely unknown. To this end, the present study was designed to capitalize on distinct fMRI modalities (11), utilizing tb-fMRI to investigate the topographical brain changes induced by NICP, while simultaneously using rs-fMRI to explore the connectivity modulations induced in the circuits of interest. During the last decades, tb-fMRI has been a widely used approach for investigating task-related networks implicated in various cognitive and motor processes (12–14). More recently, rs-fMRI has emerged as a valuable tool for investigating brain functioning in the absence of any specific task engagement (15). Particularly, rs-fMRI has been extensively utilized to explore brain functional connectivity, which refers to the temporal correlation between neurophysiological measurements obtained from distinct brain regions (16, 17). These patterns of temporally correlated oscillations observed during rest underlie the activity of the so-called resting-state networks (18, 19). Notably, Smith et al. demonstrated that these resting-state networks correspond to the same set of regions that form ‘networks,’ and are activated and/or deactivated during task performance, and provide additional means to explore further features of these neural systems (20). Remarkably, rs-fMRI data has proven valuable in predicting tb-fMRI evoked responses (21), even in pre-surgical patient populations with conditions such as tumour, epilepsy, and vascular lesions (22). Furthermore, pre-operative rs-fMRI BOLD signal is significantly affected by tumours affecting motor and language function, and associated with functionality (23). In particular, for patients with tumour near the inferior frontal gyrus, Liouta et al. found a significantly decreased rs-fMRI BOLD signal in patients with aphasia, as compared with non-aphasics, and a strong positive correlation between rs-fMRI BOLD signal and phonological fluency performance (23).

The present case report was investigated to internally validate the protocol for a subsequent (ongoing) research trial (ClinicalTrials.gov Identifier NCT05844605) (24), to verify (1) whether brain functional patterns at risk, as evidenced by tb-fMRI, could be modified through a non-invasive intensive plasticity-induction protocol; and (2), to explore the potential role of functional connectivity, assessed during rs-fMRI, as a mechanism underlying the observed changes in tb-fMRI brain activity. The main hypothesis is that the proposed neuroplasticity-promoting intervention would facilitate the establishment of new functional connections within the modulated brain system, thus facilitating the emergence of novel brain activity patterns in language network regions more distant from the tumour site. Clinically, such dualistic phenomenon (concurrent inhibition of targeted areas and enhanced recruitment of alternative resources within the same network) would result in unaltered language and cognitive performance.

## Methods

### Case description

**Figure 1:**
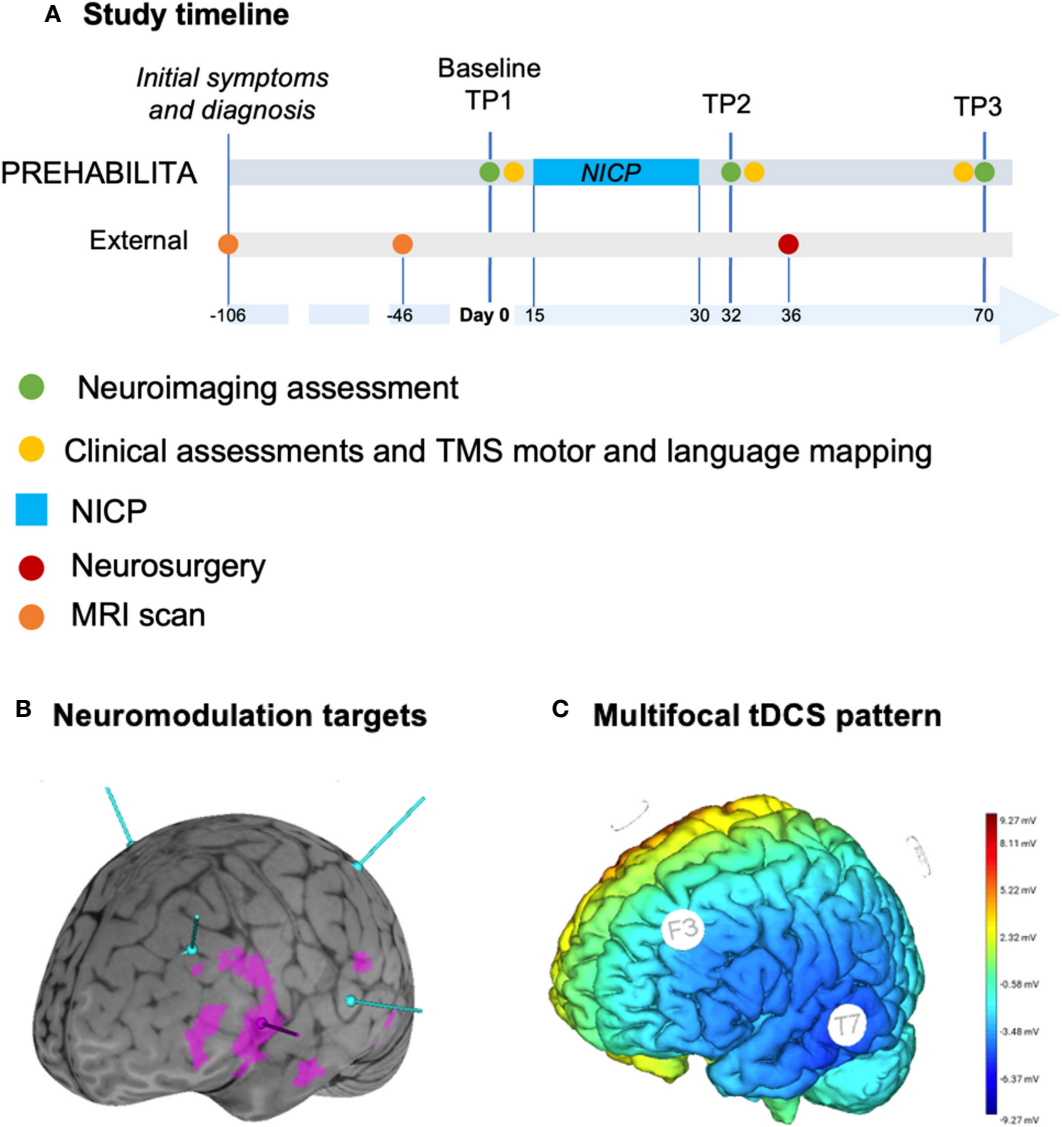
Outline of the methodology for the case report. **(A)** Study timeline, where neuroimaging assessments are depicted in green for baseline (TP1: day 0), after NICP (TP2: day 32), and after surgery (TP3: day 70). NICP (azure) consisted of 20 consecutive sessions performed between day 15 and day 30. Clinical assessments and TMS motor and language mapping (yellow) were performed at day 13, 34, and 69. Neurosurgery was performed at day 36. Previous timepoints are initial symptoms, MRI scan and diagnosis at day -106, previous MRI scan at day -46. Referral by neurosurgeon to be included in the NICP protocol was six days before baseline. **(B)** Brainsight curvilinear brain (grey) and activation clusters (derived from tb-fMRI analyses) overlay for semantic decision (violet), with targets for TMS (peak fMRI for semantic decision, in violet) and multichannel tDCS (azure) corresponding to F3, P3, T7, C4 EEG electrodes. **(C)** Map of multichannel tDCS project on Neuroelectrics software.

The patient is a right-handed adult in the 40s with past medical history reporting episodic alterations of consciousness, suggestive of epileptic seizures. During such episodes there was no relaxation of sphincters, and the patient recovered ad integrum after each episode; symptomatology presented for approximately three years. By the time of enrolment in the study protocol, no focal neurological symptoms nor clinically relevant sensorimotor or cognitive deficits were evidenced. Brain MRI demonstrated a large infiltrative lesion in the left frontobasal, temporal, and insular regions (See **Figure 1A**). At this stage, the patient was referred to Institut Guttmann (Guttmann Barcelona, Spain) from the Neurosurgery Department of Vall d’Hebron Hospital (Barcelona, Spain) to be enrolled as a voluntary participant in the PREHABILITA feasibility trial (see individualized prehabilitation description at paragraph 4.3). At the end of NICP protocol, based on clinical and MRI outcomes, a left frontotemporoparietal craniotomy was performed, and a resection of the left frontotemporoinsular space-occupying lesion (transcortical approach) was carried out without complications. Considering the size of the tumour and consequent mass effect, the neurosurgeon (F.M.R.) planned initially a two-step approach: during the first surgery intratumoural debulking was performed with the patient under general anaesthesia. Intraoperative monitoring included continuous recording from a grid of electrodes placed over the motor cortex, and by monopolar stimulation to identify the motor pathway at cortical-subcortical level. Intraoperative neuroimaging comprised neuronavigation, cerebral echography and neuronavigated echography. As planned, subtotal resection was performed, the two most limiting factors being the tumour size and associated mass effect, and the infiltration of basal ganglia at the level of perforating arteries. Based on postsurgical histopathology results (diagnosis of a grade IV frontotemporoinsular glioma with an IDH mutation), the neurosurgeon decided to cancel the second surgery (with patient awake, for further tumour removal), and instead opted for conservative patient’s management including radiotherapy and oral chemotherapy.

All procedures from the present study were performed in accordance with the Helsinki declaration. The study was approved by the Research Ethical Committee of Fundació Unió Catalana d’Hospitals (approval number: CEI 21/65, version 1, 13/07/2021).

### Assessment protocol

According to the study protocol, the patient received a comprehensive clinical, neurophysiological (i.e., TMS) and neuroimaging assessment. The same assessment was conducted at baseline (TP1, before NICP), at the end of the prehabilitation intervention (TP2, after NICP), and after neurosurgical intervention (TP3, after surgery). The feasibility of the intervention was assessed at TP2 by considering adherence to planned sessions, absence of adverse events attributable to the intervention, and patient’s satisfaction of the treatment received (PATSAT questionnaire) (25). The current case report is focused on neuropsychological and neuroimaging procedures.

To ensure transparency and reproducibility of the methods, full protocol description have been previously published (24). Further details of clinical evaluations, neuroimaging acquisition and specific processing for the present case report are available as **Supplementary Materials**.

### Individualized prehabilitation intervention

**Figure 2:**
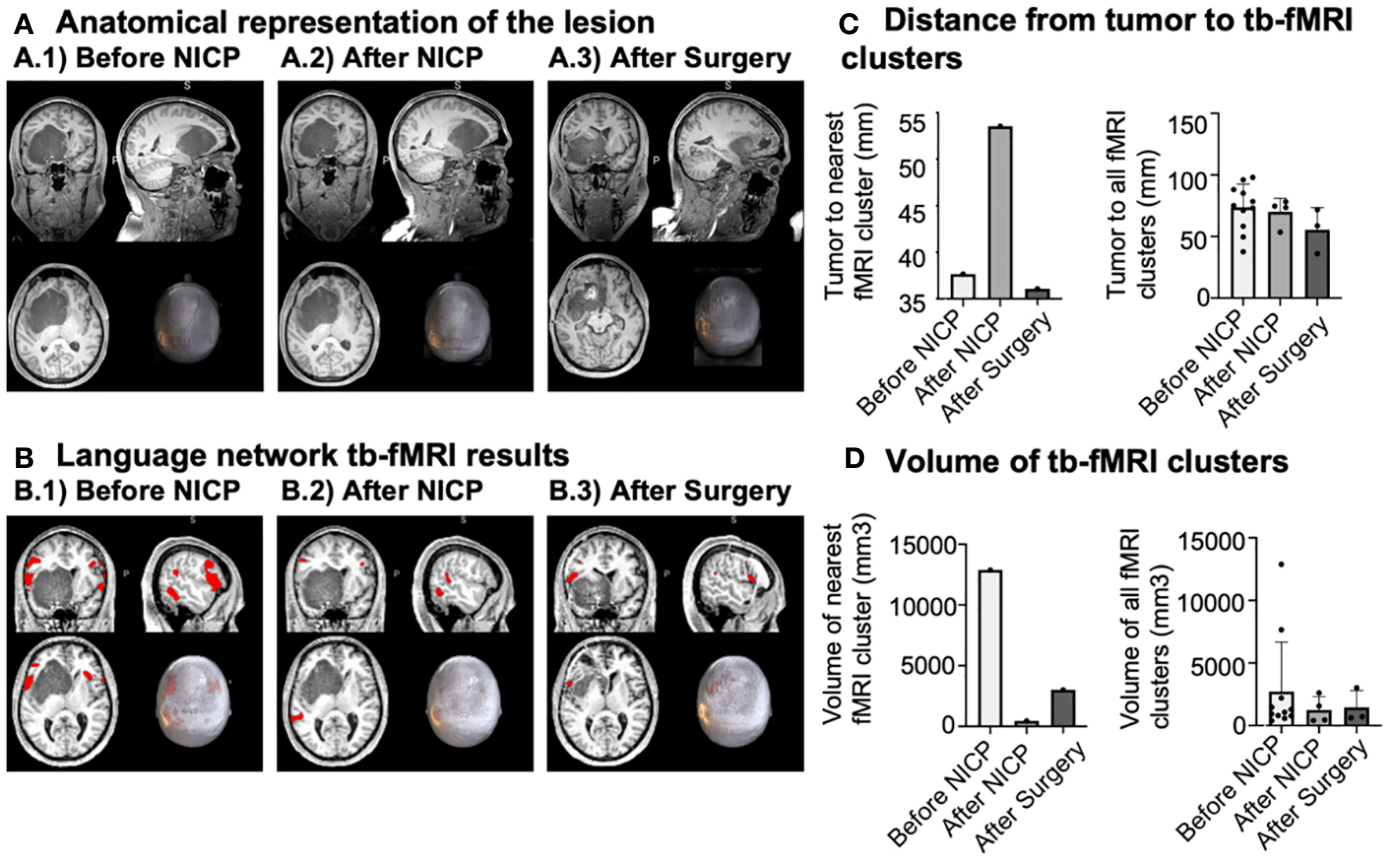
Illustration of brain tumour lesion and language network tb-fMRI results. **(A)** Anatomical representation of the tumour lesion at the three different time-points, with the centre of the figure positioned around the centre of masses. **(B)** Language network tb-fMRI results at the three different time-points, with the centre of the figure placed over the stimulation site. **(C)** Distance from the tumour to tb-fMRI clusters, presenting all tb-fMRI clusters and the nearest fMRI cluster. **(D)** Volume of tb-fMRI clusters, displaying the nearest one from the tumour and all of them.

**Figure 1** shows the timeline of interventions (A), neuromodulation targets with respect to tb-fMRI cortical activation clusters (B), and multifocal tDCS pattern (C). **Figure 2A** illustrates structural MRI of lesion distribution.

The patient performed a total of 20 sessions of NICP within 12 days, primarily organized with a first session in the morning (from 9:00 to 11:00) and a second session in the afternoon (from 14:00 to 16:00). This schedule was designed to reach the goal of at least 10 and maximum 20 sessions of NICP. Each NICP session consisted of neuromodulation coupled with intensive behavioural training. For this specific case, the function at risk of being compromised was language production. Therefore, the goal of NICP was to inhibit eloquent areas associated with language function (by means of neuromodulation) while at the same time promoting the activation of alternative nodes of the same network (by means of intensive language and cognitive training).

The neuromodulation strategy was designed to induce twofold objectives. The first aim was to achieve a focal disruption of the maximum representativity within the semantic language activation cluster identified by fMRI. The second objective was to achieve widespread inhibition across the entire semantic fMRI circuit in the left hemisphere, using parameters effective in inducing language network changes in healthy subjects and patients with aphasia (26, 27). Each morning session consisted of low frequency rTMS (1800 pulses, 1 Hz, 90% RMT) (28, 29) over the peak fMRI activation (MNI coordinates: -56, 12, 8) of the targeted cluster for semantic decision task, followed by one hour of intensive training of language and high cognitive functions with an experienced neuropsychologist (A.R.V.). The rationale for target selection was that, among the three language-related fMRI tasks, semantic decision showed the largest activation cluster, which was also the closest one to the tumour; hence, peak fMRI activation for this cluster was considered as target because of both its functional relevance and the risk of compromission by neurosurgery. Notably, if only one session per day were performed, this morning protocol was applied. Each afternoon session consisted of multifocal tDCS (30, 31) (F3: -400 μA; T7: -300 μA; P3:-300 μA; C4: 1000 μA). The main goal was to promote a widespread left inhibition over the representation of language related clusters. The total duration of tDCS sessions was 30 minutes. After the first five minutes at rest, for the remaining 25 minutes the patient received tDCS while performing intensive cognitive training by means of a dedicated online platform (Guttmann NeuroPersonalTrainer^®^, GNPT) (32). At the end of tDCS the patient performed other 30 minutes of cognitive training with GNPT, totalling approximately one hour of training. At the end of the last daily NICP session, the patient performed a High Intensity Interval Training (HIIT) protocol on a stationary bike, with the following protocol: 5 minutes warm-up, first HIIT bout (30 seconds all-out + 30 seconds rest, 10 times), 5 minutes rest, second HIIT bout (same as the first bout), 5 minutes cool-down. The goal of intensive aerobic training after cognitive training was to foster skill learning encoding and consolidation (33–35).

## Results

### Neuropsychological results

There were no significant alterations detected in any of the language tasks, which was the cognitive function of interest, at the three distinct time points pre-intervention, post-intervention, and at follow-up (i.e., TP1, TP2 and TP3; **Table 1**). Notwithstanding, during the comprehensive cognitive evaluation, a decrease in attentional, delayed memory and executive functions performance was observed, yielding clinically significant findings (which were not significant in the baseline NICP assessment). Processing speed, immediate memory and some executive function tasks were below expectation from baseline considering age and education (see **Supplementary Table 1**).

**Table 1:** Language tasks from neuropsychological assessment.

Language domain	Before NICP	After NICP	After Surgery
Spontaneous language			
Conversation Narrative speech Picture description	8	8	8
	6	6	6
	6	6	6
Informative content of language			
Fluency and grammar Content of language	10	10	10
	10	10	10
Verbal repetition			
Words Syllables Pseudowords Sentences	10	10	10
	8	8	8
	8	8	8
	60	60	60
Verboverbal Naming			
Confrontation naming Responsive naming	6	6	6
	6	6	6
Verbal comprehension			
Commands Complex ideational sentence	16	16	16
	9	9	9
Reading comprehension			
Sentences and text Word-picture Words Pseudowords	8 6 6 6	8 6 6 6	8 6 6 6
Automatic language			
Automatized sequences: Forward series Mental control: Backward series	3	3	3
	3	3	3
Naming visuoverbal			
Naming pictures	14	14	14
Reading - verbalization			
Letters Numbers Pseudowords Text	6 6 6 56	6 6 6 56	6 6 6 56
Writing: Dictate			
Letters Numbers Pseudowords Words Sentences	6 6 6 6 13	6 6 6 6 13	6 6 6 6 13

### Language tb-fMRI results

Prehabilitation resulted in an increment of the distance between the tumour (**Figure 2A**) and the nearest activation cluster during the semantic language fMRI task by 15.9 mm, returning to a similar distance as baseline after surgery (**Figures 2B, C**). Further, the volume of the closest activation fMRI cluster decreased after prehabilitation in 12.4 mm^3^, also showing a subsequent increment to a certain degree following surgery (**Figures 2B-D**).

### Language rs-fMRI network results

**Figure 3:**
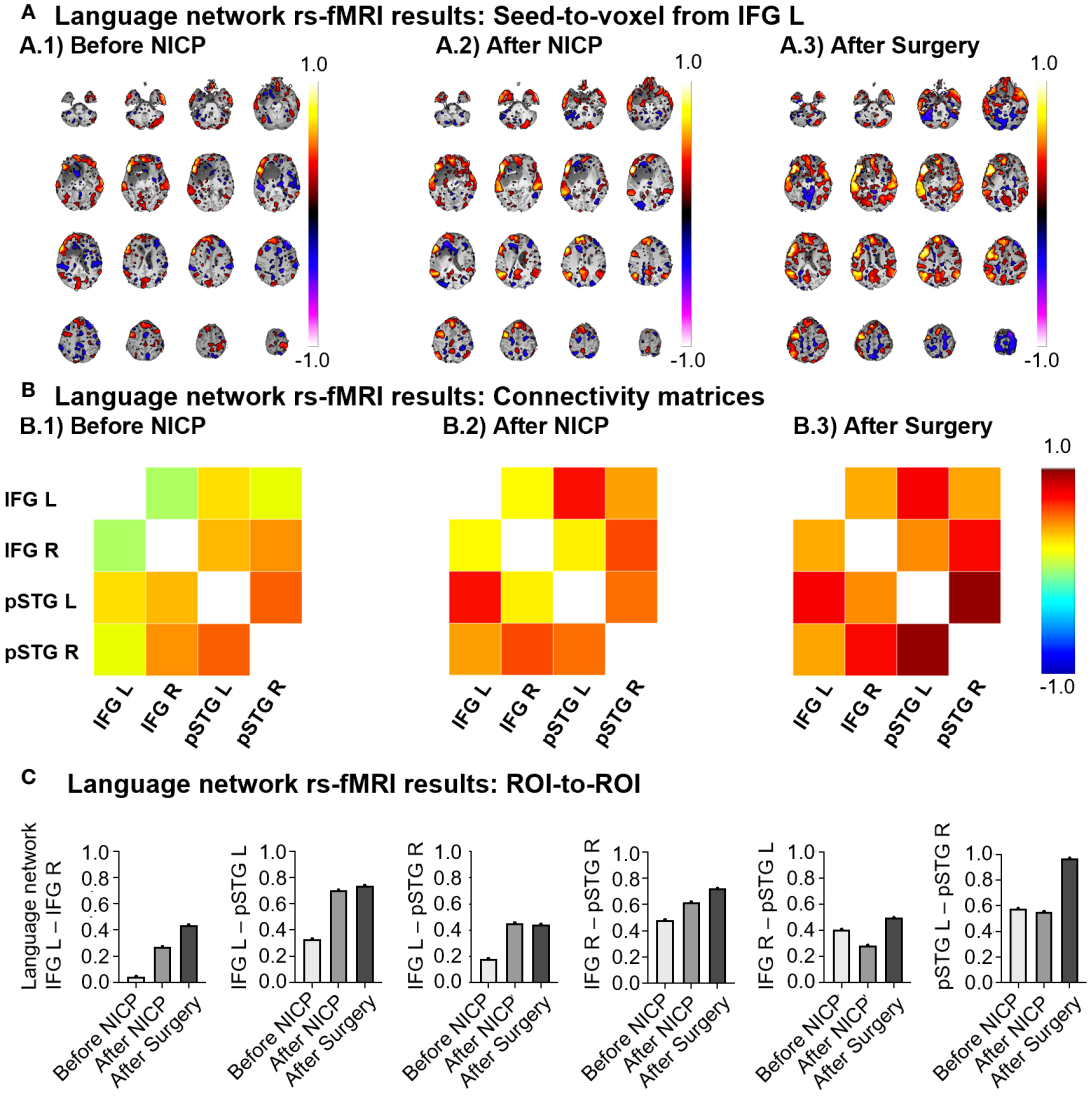
Representation of the language network rs-fMRI results. IFG L, left inferior frontal gyrus; IFG R, right inferior frontal gyrus; pSTG L, left posterior superior temporal gyrus; pSTG R, right posterior superior temporal gyrus. **(A)** Seed-to-voxel results displayed from the left inferior frontal gyrus (IFG L) at three different time-points. The colour-map represents the connectivity strength, ranging from 1 to -1. The slices are ordered along the Z-axis, ranging from -46 to 74 in increments of 8 units. **(B)** Connectivity matrices considering the four network ROIs at the three time-points. The color-map also represents the connectivity strength, ranging from 1 to -1. ROI-to-ROI results encompassing all the network couplings at the three time-points. **(C)** Histograms representing the same correlation values reported in the connectivity matrices. Each graph shows the evolution of connectivity for a specific ROI-to-ROI.

There was a noteworthy increase in the resting-state functional connectivity within the language network. This enhancement was particularly prominent between the left inferior frontal gyrus (IFG L, the nearest network node to the target of NICP neuromodulation) and the remaining regions of the language network. Remarkably, this pattern on increased connectivity persisted following the surgical procedure (**Figure 3**). In addition, an increase of functional connectivity was observed also for the right inferior frontal gyrus (IFG R), though to a lesser extent. Notably, no comparable network reconfigurations were observed within the control visual network (**Supplementary Figure 1**).

In terms of feasibility, the patient attended all planned sessions and did not report any adverse event during the whole intervention period. Results from questionnaire about patient’s satisfaction were excellent.

## Discussion

The present case report described clinical outcomes and neural correlates of a patient undergoing a non-invasive NICP protocol before neurosurgery for brain tumour. Clinically, the patient exhibited complete functionality at baseline despite the significant tumour mass and did not show clinically relevant changes for language function at the end of NICP nor at follow up after surgery (i.e., he was stable throughout the course of the intervention). On the other hand, when looking at the brain level, the patient presented baseline tb-fMRI activation clusters related to semantic decision in proximity with the tumour, particularly within the IFG L, pars opercularis. These clusters dissipated after prehabilitation with the concomitant enlargement of left temporoparietal fMRI-related clusters (specifically within the posterior divisions of the supramarginal and superior temporal gyri). Finally, after surgery, the activation clusters reappeared at approximately the same location as at baseline. Such brain activity changes were paired by resting-state functional connectivity outcomes, showing increased language network connectivity, particularly in an anteroposterior manner and mostly evident from the IFG L resting-state networks language node (with the centre coordinates over the IFG L, pars triangularis).

Despite full independence in activities of daily living, the patient presented at baseline with scores in cognitive domains such as memory and executive function below what expected based on age and education, which further decreased at the end of the intervention. Being a single case it is only possible to draw causal inferences by considering both the intervention and the tumour itself. In the first hypothesis, it’s worth considering that rTMS was applied to peak-fMRI of semantic language network and paired with speech training, while multichannel tDCS was applied to a broad parietofrontal region and paired with cognitive training. Because of the concurrent application of both modalities, it is impossible to discriminate the role of each intervention, though it would be interesting to compare the effectiveness of different approaches (TMS versus tDCS) in future studies. In the second hypothesis, the presence of the lesion determined cognitive scores already below expectations at baseline, with further worsening due to disease progression.

A critical aspect for the whole intervention was the rationale leading to the choice of the target of neuromodulation. Previous cases of non-invasive NICP selected the target based on a combination of clinical symptoms, neuroanatomical considerations, and neural correlates. Barcia et al. applied neuromodulation over a region corresponding to Broca’s area because of the proximity with the tumour and symptoms of speech disorders (6). Similarly, Dadario et al. selected targets close to the tumour and in proximity with the planned surgical entry point (10); furthermore, based on rs-fMRI results, areas that were considered hyperconnected or eligible for excision were inhibited, and areas hypoconnected or potential candidates to functionally supply eloquent areas were stimulated (with excitatory paradigms). When looking at invasive NICP case reports (7, 9) and case series (8), a common element was the placement of grids of electrodes for the application of cortical electrical stimulation at the maximum tolerable intensity; grids were placed over extended regions covering eloquent areas, based on clinical and neuroanatomical considerations. For the present study, the patient was completely functional at baseline, hence the starting point was considering the anatomical localization of the tumour, the cortical distribution of language tb-fMRI clusters, and the localization of the peak-fMRI for each cluster of interest. By delivering a regional neuromodulation, the goal was to elicit widespread neuroplastic reorganization. In this perspective, peak tb-fMRI of the largest cluster close to the tumour was selected as the centre of a relevant node within the semantic language network and targeted with low frequency rTMS to induce a topographical rearrangement of this brain circuit. Furthermore, inhibitory multifocal tDCS was applied with cathodes mainly covering the identified circuit of interest, to boost the inhibition within the targeted cluster in favour of other compensatory network areas.

When looking at functional activation associated to semantic decision task, the minimum distance between the tumour and any activation peak increased by almost 16 mm from TP1 to TP2, indicating an antero-posterior shift of functional activity (i.e., from frontal to the temporoparietal brain regions). From a neurosurgical perspective, a distance between a lesion and eloquent area less than 5 mm is associated with worse outcomes (36, 37). Therefore, the increase in minimum distance obtained may be considered of direct clinical relevance. Furthermore, the minimum distance between the tumour and peak-fMRI returned to approximately baseline levels at TP3, hence suggesting that non-invasive NICP provoked a temporal window of neuroplastic changes beneficial for the preservation of functionality during neurosurgery; in the absence of any specific treatment and likely following spontaneous recovery, the brain reorganized itself by returning to a pattern of functional activity comparable to what was evidenced before the intervention.

When focusing on rs-fMRI, seed-based analysis revealed a notable functional connectivity increase within the language resting-state networks. Specifically, ROI-to-ROI analyses showed an increased connectivity between the IFG L area and all other nodes in the network. Additionally, an anteroposterior connectivity increment between the right hemisphere’s IFG and the posterior superior temporal gyrus (pSTG) was observed. Importantly, these functional connectivity patterns were not present in a control visual network. Therefore, our NICP protocol enhanced the rs-fMRI connectivity of the language network, with a main emphasis on the IFG L, the node roughly aligned with the tb-fMRI peak activation used as the targeted TMS area, and particularly in an anteroposterior fashion. Interestingly, the rs-fMRI functional connectivity results for the language network are spatially consistent with the findings from semantic language tb-fMRI outcomes, wherein there was a subsequent reduction of clusters anteriorly, at the level of the IFG L, and the enlargement of clusters in posterior areas of the network, within the temporoparietal intersection. Consequently, it appears that our NICP protocol was capable of modulating both tb-fMRI brain activity and rs-fMRI functional connectivity. More precisely, this modulation resulted in an amplification of rs-fMRI functional connectivity within the language system, which might presumably underlie the subsequent displacement of brain activation to other regions, farther from the lesion, during task demands.

Some limitations should be addressed. First, the present study is a case report, which heavily limits the interpretation and generalizability of findings, warranting future group-level studies. Another important constraint is that the patient underwent a complex intervention, composed of two different protocols of neuromodulation (low frequency rTMS and multifocal tDCS) coupled with intensive language and cognitive training, followed by intensive aerobic training to promote the consolidation of neuroplastic changes. The overall rationale was to provide a comprehensive intervention based on the best available evidence to achieve the most ambitious clinical outcome, tailored to specific patient’s needs. Nonetheless, this prevents us from determining the relative contribution of each ingredient on the outcome of the intervention. Future comparative studies may help elucidating this aspect. Finally, in the absence of a control condition, it is not possible to determine to which extent neuroplastic changes were due to the intervention. Indeed, the presence of the tumour itself may significantly affect the coupling between neural activity and blood flow (neurovascular uncoupling), possibly jeopardizing the interpretation of functional neuroimaging outcomes (38). However, data from the present case indicates a shift of the cortical activation pattern within nodes of the language network, suggesting a true neuroplastic reorganization rather than a random artifact. In conclusion, when putting the present study in perspective with previous literature, it is important to acknowledge that this is the first case showing clinically relevant neuroplastic changes after non-invasive NICP coupled with intensive task training without neurological sequelae. Hence, non-invasive NICP holds significance as an attractive alternative to invasive NICP protocols, warranting further investigation.

## Data availability statement

The datasets presented in this article are not readily available because of ethical and privacy restrictions. Requests to access the datasets should be directed to the corresponding authors.

## Ethics statement

The studies involving humans were approved by Fundació Unió Catalana d’Hospitals (approval number: CEI 21/65, version 1, 13/07/2021). The studies were conducted in accordance with the local legislation and institutional requirements. The participants provided their written informed consent to participate in this study. Written informed consent was obtained from the individual(s) for the publication of any potentially identifiable images or data included in this article.

## Author contributions

LB: Conceptualization, Data curation, Formal analysis, Investigation, Methodology, Project administration, Software, Writing – original draft, Writing – review & editing. AR: Investigation, Validation, Writing – original draft, Writing – review & editing. EB: Investigation, Project administration, Writing – original draft, Writing – review & editing. DL: Investigation, Writing – review & editing. JM: Writing – review & editing. MC: Data curation, Formal analysis, Investigation, Methodology, Software, Writing – original draft, Writing – review & editing. RP: Conceptualization, Software, Writing – review & editing. JP: Data curation, Formal analysis, Writing – review & editing, Validation. CL: Formal Analysis, Writing – review & editing, Validation. CG: Resources, Writing – review & editing. EM: Methodology, Resources, Writing – review & editing. NB: Methodology, Supervision, Writing – review & editing. GV: Investigation, Resources, Writing – review & editing. FM: Resources, Writing – review & editing. CT: Investigation, Writing – review & editing. LM: Investigation, Writing – review & editing. MS: Writing – review & editing. DB: Funding acquisition, Methodology, Project administration, Supervision, Writing – review & editing. KA: Formal analysis, Investigation, Methodology, Project administration, Software, Supervision, Writing – original draft, Writing – review & editing. AP: Conceptualization, Funding acquisition, Project administration, Supervision, Writing – review & editing. JT: Conceptualization, Data curation, Funding acquisition, Investigation, Project administration, Supervision, Writing – review & editing.
