# Supplementary material for: Exploring the neural basis of non-invasive prehabilitation in brain tumour patients: An fMRI-based case report of language network plasticity
Source: Front Oncol. 2024 May 17;14:1390542. doi: 10.3389/fonc.2024.1390542 (PMC11140081; doi:10.3389/fonc.2024.1390542)
Supplement: Supplementary file 1 [file DataSheet_1.pdf]

SUPPLEMENTARY MATERIAL

Exploring the neural basis of non-invasive prehabilitation in brain tumour patients: An fMRI-based case report of language network plasticity.

Leonardo Boccuni[1,2,3], Alba Roca-Ventura[1,4,5], Edgar Buloz-Osorio[1,2,4], David Leno-Colorado[1,2,4], Jesús Martín-Fernández[6,7,8], María Cabello-Toscano[5,9], Ruben Perellón-Alfonso[1,2,4,5,9], José Carlos Pariente[9,10], Carlos Laredo[9,10], Cesar Garrido[9,10,11], Emma Muñoz-Moreno[9,10], Nuria Bargalló[5,9,10,11,12], Gloria Villalba[13], Francisco Martínez-Ricarte[14], Carlo Trompetto[15,16], Lucio Marinelli[15,16], Matthew D. Sacchet[17,18], David Bartrés-Faz[1,5,9], Kilian Abellaneda-Pérez[1,2,4]†*, Alvaro Pascual-Leone[1,19,20]†, Josep María Tormos Muñoz[1,21]†*

1 Institut Guttmann, Institut Universitari de Neurorehabilitació adscrit a la UAB, Badalona, Barcelona, Spain.

2 Universitat Autònoma de Barcelona, Bellaterra, Cerdanyola del Vallès, Spain.

3 Scientific Institute IRCCS E. Medea, Department of Conegliano, Italy.

4 Fundació Institut d'Investigació en Ciències de la Salut Germans Trias i Pujol, Badalona, Barcelona, Spain.

5 Departament de Medicina, Facultat de Medicina i Ciències de la Salut, Institut de Neurociències, Universitat de Barcelona, Spain.

6 Department of Neurosurgery, Hôpital Gui de Chauliac, Montpellier, France.

7 Department of Neurosurgery, Hospital Universitario Nuestra Señora de Candelaria, Tenerife, Spain.

8 Universidad de La Laguna, Tenerife, Spain.

9 Institut d'Investigacions Biomèdiques August Pi i Sunyer (IDIBAPS), Barcelona, Spain.

10 Magnetic Resonance Image Core Facility (IDIBAPS), Barcelona, Spain.

11 Neuroradiology Section, Radiology Department, Diagnostic Image Centre, Hospital Clinic of Barcelona, University of Barcelona, Barcelona, Spain.

12 Centro de Investigación Biomédica en Red de Salud Mental (CIBERSAM), Instituto de Salud Carlos III, Barcelona, Spain.

13 Department of Neurosurgery, Hospital del Mar, Barcelona, Spain.

14 Department of Neurosurgery, Vall d'Hebron Hospital, Universitat Autònoma de Barcelona, Barcelona, Spain.
15 University of Genova, Department of Neuroscience, Rehabilitation, Ophthalmology, Genetics, Maternal and Child Health, Genova, Italy.
16 IRCCS Ospedale Policlinico San Martino, Genova, Italy.
17 Meditation Research Program, Department of Psychiatry, Massachusetts General Hospital, Harvard Medical School, Boston, MA, USA
18 Athinoula A. Martinos Centre for Biomedical Imaging, Department of Radiology, Massachusetts General Hospital, Harvard Medical School, Charlestown, MA, USA
19 Hinda and Arthur Marcus Institute for Aging Research and Deanna and Sidney Wolk Centre for Memory Health, Hebrew Senior Life, Boston, MA, USA.
20 Department of Neurology, Harvard Medical School, Boston, MA, USA.
21 Centro de Investigación Traslacional San Alberto Magno, Universidad Católica de Valencia San Vicente Mártir, Valencia, Spain.

* Correspondence: K.A.P.: kabellaneda@guttmann.com; Tel.: +34-93-497-7700. J.M.T.: jm.tormos@ucv.es; Tel.: +34-68-694-0393
† These authors contributed equally to this work.

1. Methods - supplementary

1.1 Neuropsychological evaluation

The different tests used to evaluate cognitive domains are detailed below: (1) For the clinical assessment of language, several subtests from the Barcelona Test Revised (TB-R)(1) were used to evaluate spontaneous and automatic language, repetition, naming, verbal comprehension, reading, reading comprehension, and writing; (2) Orientation (person, space, time) was assessed using the orientation subtest of the TB-R(1); (3) Attention was evaluated using the direct digit subtest of the WAIS-III(2), and the Trail Making Test A and B (TMT A, TMT B)(3); (4) Verbal memory was assessed using the Rey Auditory Verbal Learning Test (RAVLT)(4); (5) The Symbol Digit Modalities Test (SDMT)(5) was used to assess processing speed; (6) Visuoperceptual skills were assessed using the overlapping figures subtest, and visuoconstructive skills were assessed using the visual-graphic attention subtest, both from the TB-R(1); (7) Praxis was assessed using

the symbolic gesture to command and imitation of postures subtests from the TB-R(1); (8) Executive functions were evaluated using the letter-number sequencing subtest, digit span backward subtest, and block design subtest from the WAIS-III(2), verbal fluency by letters PMR(6), Hayling Test(7), Arithmetic and Mental Control from the TB-R(1), and Wisconsin Card Sorting Test (WCST)(8).

1.2 Neuroimaging acquisition

MRI data were acquired in a 3T Siemens scanner (MAGNETOM Prisma) with 64-channel head coil at Magnetic Resonance Imaging Core Facility at IDIBAPS (Institut d'Investigacions Biomèdiques August Pi i Sunyer), Barcelona, Spain. The same imaging protocol was acquired at the three time points including 3D structural T1-weighted image followed by a rs-fMRI and 8 different tb-fMRI. High-resolution T1-weighted structural image was obtained with a magnetization prepared rapid acquisition gradient-echo (MPRAGE) three-dimensional protocol (repetition time (TR) = 2,400 ms, echo time (TE) = 2.22 ms, inversion time = 1,000 ms, field of view (FOV) = 240x256 mm^2, flip angle = 8º and 0.8 mm^3 isotropic voxel). The acquisition parameter for functional imaging were the same for both resting and task-based protocols: anterior-posterior phase-encoding; acceleration factor = 8, interleaved acquisition, T2*weighted EPI scans, TR = 800 ms (750 volumes, total time for acquisition = 10 minutes), TE = 37 ms, 72 slices, slice thickness = 2 mm, FOV = 208x208 mm^2, voxel-size=2x2x2 mm^3). Tb-fMRI was acquired during three language and five motor paradigms(9–11), which adhered to the following procedures: (1) Word generation task: Blocks paradigm consisting in five cycles. Each cycle comprises 30 seconds of rest followed by 30 seconds during which the participant must mention words starting with a certain letter. These letters are "F", "A", "S", "M" and "E", in this same order. (2) Semantic decision task: Blocks paradigm consisting in five cycles. Each cycle comprises 30 seconds of rest followed by 30 seconds during which the participant must think about objects from certain places: school, kitchen, car, house, and hospital. (3) Comprehensive auditive task: Blocks paradigm consisting in three cycles. Each cycle comprises a 30 second block, in which a story is narrated in Spanish (active block/condition), followed by another 30 seconds block, in which the language content of the story is reproduced digitally backwards (inactive/rest block/condition). After the acquisition, the patient was asked about the story to control their performance in the task. This was an fMRI adaptation of the logical memory test of the Wechsler

Memory Scale-3rd Edition(12). Motor-related fMRI tasks (i.e., bilateral finger tapping task, bilateral ankle flexion task, and tongue movement task) were acquired as part of the standardized tb-fMRI protocol, although for this case study they were not deemed as clinically relevant as language-related fMRI tasks.

1.3 Neuroimaging analysis

Imaging data analysis and visualization was performed using FMRIB's Software Library (FSL, version 6.0.5.1; http://fsl.fmrib.ox.ac.uk/fsl)(13–15), Statistical Parametric Mapping (SPM, version 12; https://www.fil.ion.ucl.ac.uk/spm/)(16), CONN Toolbox (release 22.a, https://web.conn-toolbox.org/)(17,18), MRIcroGL (version 12.6; https://www.nitrc.org/projects/mricrogl/)(19), ITK Snap (version 3.8.0; http://www.itksnap.org)(20), GraphPad Prism (version 9.0.0; https://www.graphpad.com/)(21), and MATLAB (versions R2022a and 2023a; https://www.mathworks.com/)(22).

1.3.1 Lesion segmentation

Native structural MRI was first normalized to Montreal Neurology Institute (MNI) standard space; subsequently, the brain tumour of the patient was semi-automatically segmented at distinct MRI time-points (i.e., TP1, TP2, TP3) using the ITK-SNAP software by a physician with extended knowledge in neuroanatomy (E.B.-O.). The resulting segmentation was thoroughly examined and, if necessary, refined to ensure precise delineation of the tumours.

1.3.2 Task-based fMRI

1.3.2.1 Preprocessing

Tb-fMRI data was analysed using an in-house developed toolbox (MAGIC fMRI toolbox(23)), based on statistical parametric mapping SPM12(16)). First, fMRI was pre-processed including motion correction, slice-time correction, and correction of EPI distortion by elastic registration to structural T1-weighted image. Then, the images were normalized to MNI (2 mm isotropic voxel) standard space by non-linear registration to the template. Finally, a Gaussian spatial smoothing (8 mm full width at half maximum) was applied.

Activation maps were generated for the task conditions by fitting a general linear model voxel-wise to the pre-processed fMRI images. The canonical hemodynamic response function was convolved with a boxcar function to represent condition-specific changes in the signal. To minimize residual errors, previously estimated motion parameters were included as nuisance regressors in the design matrix. The contrast of interest was calculated by subtracting the condition of interest (i.e., semantic decision) from the rest condition (baseline). Statistical significance was determined using a threshold of $p < 0.001$ and a family-wise error cluster threshold of 0.01.

1.3.2.2 First-level analysis

To model brain activation of each task condition, general linear models were fitted voxel-wise. In these, the canonical hemodynamic response function and a boxcar function were convolved together to model condition-specific changes on the signal. Previously estimated motion parameters were regressed out in these models. Then, subtraction of rest from tasks conditions was calculated. The resulting maps comprising activated areas were considered as statistically significant when p-value was under 0.001 and multiple comparisons were applied on a family-wise error cluster threshold of p-value under 0.05.

1.3.3 Resting-state fMRI

1.3.3.1 Preprocessing

The first ten functional images were discarded to reduce scanner inhomogeneity. Then, functional and anatomical data were pre-processed using a flexible preprocessing pipeline(24) including realignment with correction of susceptibility distortion interactions, outlier detection, direct segmentation and MNI-space normalization, and smoothing. Functional data were realigned using SPM realign and unwarp procedure(25), where all scans were coregistered to a reference image (first scan of the first session) using a least squares approach and a 6 parameter (rigid body) transformation(26), and resampled using b-spline interpolation to correct for motion and magnetic susceptibility interactions. Potential outlier scans were identified using ART(27) as acquisitions with framewise displacement above 0.9 mm or global BOLD signal changes above 5 standard deviations(28,29), and a reference BOLD image was computed for each subject by averaging all scans excluding outliers. Functional and anatomical data were normalized into standard MNI space, segmented into grey matter, white matter, and cerebrospinal fluid (CSF) tissue classes, and resampled to 2 mm isotropic voxels following a direct

normalization procedure(29,30) using SPM unified segmentation and normalization algorithm(31,32) with the default IXI-549 tissue probability map template. Last, functional data were smoothed using spatial convolution with a Gaussian kernel of 6 mm full width half maximum.

1.3.3.2 Denoising

In addition, functional data were denoised using a standard denoising pipeline(33) including the regression of potential confounding effects characterized by white matter timeseries, CSF timeseries, motion parameters and their first order derivatives(34), outlier scans(28), and linear trends within each functional run, followed by bandpass frequency filtering of the BOLD timeseries(35) between 0.008 Hz and 0.09 Hz. CompCor(36) noise components within white matter and CSF were estimated by computing the average BOLD signal as well as the largest principal components orthogonal to the BOLD average, motion parameters, and outlier scans within each subject's eroded segmentation masks.

1.3.3.3 Functional connectivity analyses

Seed-based connectivity maps and ROI-to-ROI connectivity matrices were estimated characterizing the patterns of functional connectivity using 8 HPC-ICA network ROIs(37). Four ROIs belonged to the language network, and contained the following nodes: left inferior frontal gyrus (IFG L; -51,26,2), right IFG (IFG R; 54,28,1), left posterior superior temporal gyrus (pSTG L; -57,-47,15), and right pSTG (pSTG R; 59,-42,13). The rest of the ROIs pertained to the visual network, used as a control. The nodes of this network are referred as visual medial (VM; 2,-79,12), visual occipital (VO; 0,-93,-4), visual lateral left (VL L; -37,-79,10), and visual lateral right (VL R; 38,-72,13). functional connectivity strength was represented by Fisher-transformed bivariate correlation coefficients from a weighted general linear model (weighted-GLM)(38), defined separately for each pair of seed and target areas, modelling the association between their BOLD signal timeseries. To compensate for possible transient magnetization effects at the beginning of each run, individual scans were weighted by a step function convolved with an SPM canonical hemodynamic response function and rectified. Ultimately, the average connectivity within each seed-based map was computed, utilizing a threshold of 0.2, and subsequently compared across sessions.

1.3.4 Euclidean distances calculations

The Euclidean distance between the brain tumour's centre of mass coordinates and the coordinates of the activated regions identified with the tb-fMRI paradigm were calculated using an in-house MATLAB code.

2. Results - supplementary

Supplementary Table 1. Neuropsychological evaluation.

Cognitive domain	Before NICP	After NICP	After Surgery
**Orientation**			
Personal	7	7	7
Spatial	5	5	5
Temporal	23	23	23
**Attention**			
Digit Span Forward	7	6	6
TMT A - time	39	26	36
TMT B - time	64	70*	96*
**Memory**			
RAVLT Immediate	46*	55	44*
RAVLT Delayed	10	12	9*
RAVLT Recognition	14	15	13
**Processing speed**			
Digit-symbol (coding)	41*	67	62*
**Visual perception**			
Superimposed figures	20	20	20
Visuospatial attention	28	28	28
**Praxis**			
Symbolic gestures - command	20	20	20
Symbolic gestures - imitation	20	20	20
**Executive functions**			
Letter and Number sequencing	13	14	11
Digit Span Backward	6	7	4
Block design	35	37	24*
Phonological fluency (PMR)	41*	46*	33*
Hayling Test time	2,6	3,26	4,7
Hayling Test score	1,6	1,5	1,5
Arithmetic	9	9	9
WCST categories	4*	1*	/
WCST perseverative errors	37*	53*	/

Notes: (1) The asterisk denotes scores that hold clinical significance. (2) WCST categories and WCST perseverative errors were not assessed at T3.

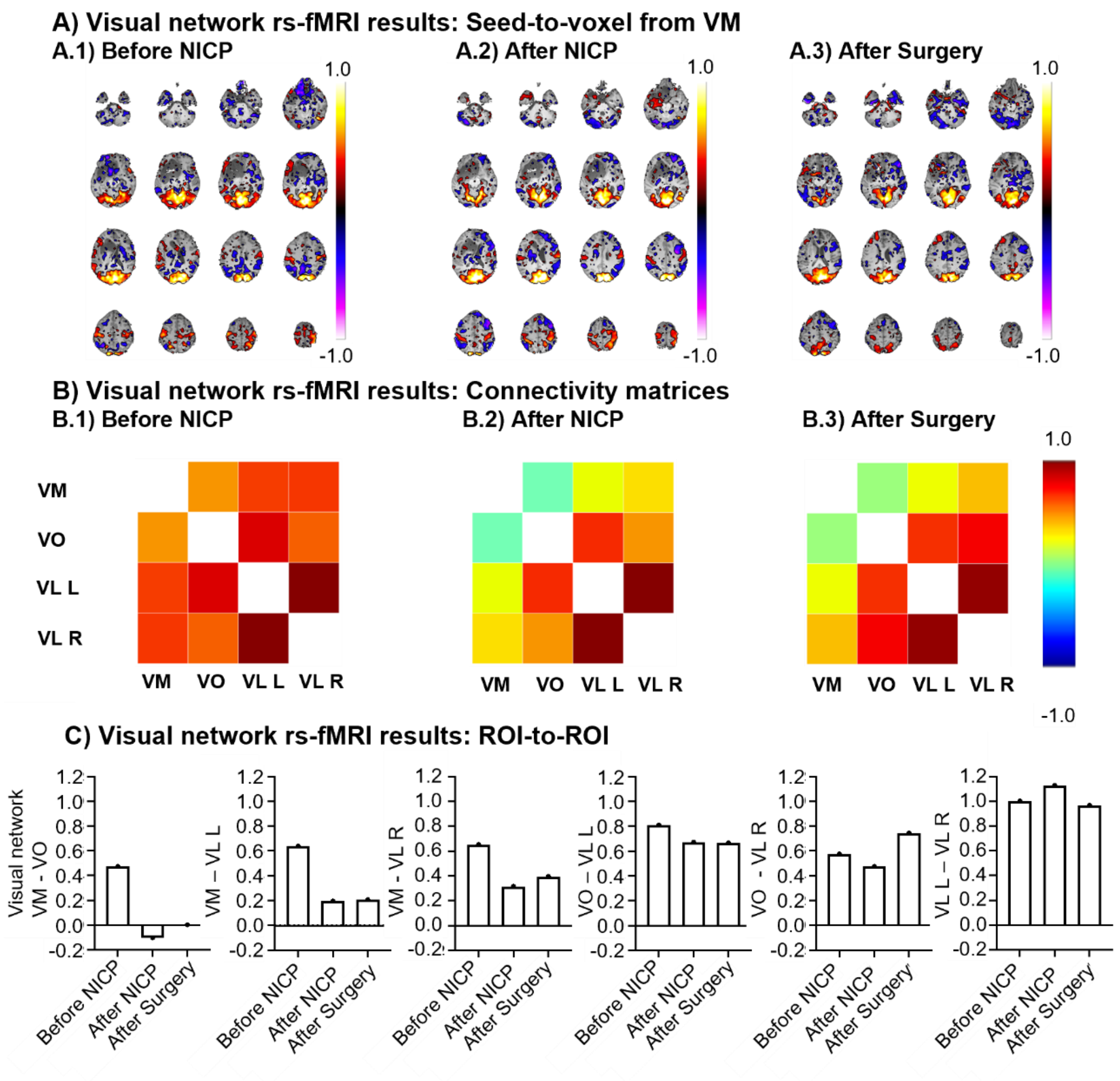

Supplementary Figure 1. Representation of the control visual network rs-fMRI results. VM, visual medial network; VO, visual occipital network; VL L, visual lateral network (left hemisphere); VL R, visual lateral network (right hemisphere). A) Seed-to-voxel results displayed from the VM at three different time-points. The color-map represents the connectivity strength, ranging from 1 to -1. The slices are ordered along the Z-axis, ranging from -46 to 74 in increments of 8 units. B) Connectivity matrices considering the four network ROIs at the three time-points. The color-map also represents the connectivity strength, ranging from 1 to -1. ROI-to-ROI results encompassing all the network couplings at the three time-points. C) Histograms representing the same correlation values reported in the connectivity matrices. Each graph shows the evolution of connectivity for a specific ROI-to-ROI.

References

1. Peña-Casanova J, Esparcia AJJ, Saladié DG i, Olmos JG. *Normalidad, semiología y patología neuropsicológicas: programa integrado de exploración neuropsicológica "Test Barcelona."* Masson (1991).

2. Cubero NS, Wechsler D. *WAIS-III: Escala de Inteligencia de Wechsler para Adultos III. Manual técnico*. TEA Ediciones (1999).

3. Reed JC, Reed HBC. "The Halstead—Reitan Neuropsychological Battery.," In: Goldstein G, Incagnoli TM, editors. *Contemporary Approaches to Neuropsychological Assessment*. Critical Issues in Neuropsychology. Boston, MA: Springer US (1997). p. 93–129 doi: 10.1007/978-1-4757-9820-3_4

4. Schmidt M. *Rey Auditory Verbal Learning Test: RAVLT : a handbook*. Los Angeles, CA: Western Psychological Services (1996).

5. Smith A. *Symbol digit modalities test*. Western psychological services Los Angeles (1973).

6. Artiola I. Fortuny L, Romo DH, Heaton RK, Pardee Iii RE. *Manual de Normas y Procedimientos Para la Bateria Neuropsicologia*. Psychology Press (1999).

7. Pérez-Pérez A, Matias-Guiu JA, Cáceres-Guillén I, Rognoni T, Valles-Salgado M, Fernández-Matarrubia M, Moreno-Ramos T, Matías-Guiu J. The Hayling Test: Development and Normalization of the Spanish Version. *Archives of Clinical Neuropsychology* (2016) 31:411–419. doi: 10.1093/arclin/acw027

8. Berg EA. A Simple Objective Technique for Measuring Flexibility in Thinking. *The Journal of General Psychology* (1948) 39:15–22. doi: 10.1080/00221309.1948.9918159

9. González-Ortiz S, Oleaga L, Pujol T, Medrano S, Rumiá J, Caral L, Boget T, Capellades J, Bargalló N. Simple fMRI postprocessing suffices for normal clinical practice. *AJNR Am J Neuroradiol* (2013) 34:1188–1193. doi: 10.3174/ajnr.A3381

10. Cano-López I, Calvo A, Boget T, Carreño M, Donaire A, Setoain X, Pintor L, Rumià J, González-Bono E, Junqué C, et al. Typical asymmetry in the hemispheric activation during an fMRI verbal comprehension paradigm is related to better performance in verbal and non-verbal tasks in patients with epilepsy. *Neuroimage Clin* (2018) 20:742–752. doi: 10.1016/j.nicl.2018.09.010

11. Bargalló N, Cano-López I, Rosazza C, Vernooij MW, Smits M, Vitali P, Alvarez-Linera J, Urbach H, Mancini L, Ramos A, et al. Clinical practice of language fMRI in epilepsy centers: a European survey and conclusions by the ESNR Epilepsy Working Group. *Neuroradiology* (2020) 62:549–562. doi: 10.1007/s00234-020-02397-w

12. Wechsler III D. *WMS-III Administration and Scoring Manual. San Antonio, TX: The Psychological Corporation*. Harcourt Brace & Co (1997).

13. Smith SM, Jenkinson M, Woolrich MW, Beckmann CF, Behrens TEJ, Johansen-Berg H, Bannister PR, De Luca M, Drobnjak I, Flitney DE, et al. Advances in functional and structural MR image analysis and implementation as FSL. *Neuroimage* (2004) 23 Suppl 1:S208-219. doi: 10.1016/j.neuroimage.2004.07.051

14. Woolrich MW, Jbabdi S, Patenaude B, Chappell M, Makni S, Behrens T, Beckmann C, Jenkinson M, Smith SM. Bayesian analysis of neuroimaging data in FSL. *Neuroimage* (2009) 45:S173-186. doi: 10.1016/j.neuroimage.2008.10.055

15. Jenkinson M, Beckmann CF, Behrens TEJ, Woolrich MW, Smith SM. FSL. *Neuroimage* (2012) 62:782–790. doi: 10.1016/j.neuroimage.2011.09.015

16. SPM - Statistical Parametric Mapping. (2023) https://www.fil.ion.ucl.ac.uk/spm/ [Accessed July 21, 2023]

17. Whitfield-Gabrieli S, Nieto-Castanon A. Conn: a functional connectivity toolbox for correlated and anticorrelated brain networks. *Brain Connect* (2012) 2:125–141. doi: 10.1089/brain.2012.0073

18. Nieto-Castanon A. Brain-wide connectome inferences using functional connectivity MultiVariate Pattern Analyses (fc-MVPA). *PLOS Computational Biology* (2022) 18:e1010634. doi: 10.1371/journal.pcbi.1010634

19. NITRC: Welcome. (2023) https://www.nitrc.org/ [Accessed July 22, 2023]

20. Yushkevich PA, Piven J, Hazlett HC, Smith RG, Ho S, Gee JC, Gerig G. User-guided 3D active contour segmentation of anatomical structures: significantly improved efficiency and reliability. *Neuroimage* (2006) 31:1116–1128. doi: 10.1016/j.neuroimage.2006.01.015

21. Home - GraphPad. (2023) https://www.graphpad.com/ [Accessed July 22, 2023]

22. MATLAB. (2023) https://www.mathworks.com/products/matlab.html [Accessed July 22, 2023]

23. Pariente JC, Pascual-Diaz S, Muñoz-Moreno E, GARRIDO C, Conde E, Ispan CA, Alabart NB. MAGIC, a user-friendly toolbox to analyze language fMRI in clinic practice. *ECR 2020 EPOS* (2020) https://epos.myesr.org/poster/esr/ecr2020/C-09248 [Accessed July 24, 2023]

24. Nieto-Castanon, A. "FMRI minimal preprocessing pipeline.," *Handbook of functional connectivity Magnetic Resonance Imaging methods in CONN*. Hilbert Press (2020). p. 3–16

25. Andersson JLR, Hutton C, Ashburner J, Turner R, Friston K. Modeling Geometric Deformations in EPI Time Series. *NeuroImage* (2001) 13:903–919. doi: 10.1006/nimg.2001.0746

26. Friston KarlJ, Ashburner J, Frith CD, Poline J-B, Heather JD, Frackowiak RSJ. Spatial registration and normalization of images. *Human Brain Mapping* (1995) 3:165–189. doi: 10.1002/hbm.460030303

27. Whitfield-Gabrieli, S., Nieto-Castanon, A., & Ghosh, S. Artifact detection tools (ART). (2011)

28. Power JD, Mitra A, Laumann TO, Snyder AZ, Schlaggar BL, Petersen SE. Methods to detect, characterize, and remove motion artifact in resting state fMRI. *Neuroimage* (2014) 84:320–341. doi: 10.1016/j.neuroimage.2013.08.048

29. Nieto-Castanon A. Preparing fMRI Data for Statistical Analysis. (2022) doi: 10.48550/arXiv.2210.13564

30. Calhoun VD, Wager TD, Krishnan A, Rosch KS, Seymour KE, Nebel MB, Mostofsky SH, Nyalakanai P, Kiehl K. The impact of T1 versus EPI spatial normalization templates for fMRI data analyses. *Hum Brain Mapp* (2017) 38:5331–5342. doi: 10.1002/hbm.23737

31. Ashburner J, Friston KJ. Unified segmentation. *Neuroimage* (2005) 26:839–851. doi: 10.1016/j.neuroimage.2005.02.018

32. Ashburner J. A fast diffeomorphic image registration algorithm. *Neuroimage* (2007) 38:95–113. doi: 10.1016/j.neuroimage.2007.07.007

33. Nieto-Castanon, A. “FMRI denoising pipeline.,” *Handbook of functional connectivity Magnetic Resonance Imaging methods in CONN*. Hilbert Press. (2020). p. 17–25

34. Friston KJ, Williams S, Howard R, Frackowiak RS, Turner R. Movement-related effects in fMRI time-series. *Magn Reson Med* (1996) 35:346–355. doi: 10.1002/mrm.1910350312

35. Hallquist MN, Hwang K, Luna B. The Nuisance of Nuisance Regression: Spectral Misspecification in a Common Approach to Resting-State fMRI Preprocessing Reintroduces Noise and Obscures Functional Connectivity. *Neuroimage* (2013) 0:208–225. doi: 10.1016/j.neuroimage.2013.05.116

36. Behzadi Y, Restom K, Liau J, Liu TT. A Component Based Noise Correction Method (CompCor) for BOLD and Perfusion Based fMRI. *Neuroimage* (2007) 37:90–101. doi: 10.1016/j.neuroimage.2007.04.042

37. Nieto-Castanon, A. & Whitfield-Gabrieli, S. CONN functional connectivity toolbox: RRID SCR_009550, release 22. (2022)

38. Nieto-Castanon, A. “Functional Connectivity measures. In Handbook of functional connectivity Magnetic Resonance Imaging methods in CONN.,” Hilbert Press. (2020). p. 26–62
